# A Case of Colorectal Cancer during Pregnancy: A Brief Review of the Literature

**DOI:** 10.1155/2013/626393

**Published:** 2013-01-14

**Authors:** Sepideh Khodaverdi, Ali Kord Valeshabad, Maryam Khodaverdi

**Affiliations:** ^1^Research Development Center, Shariati Hospital, Tehran University of Medical Sciences, P.O. Box 14155-6447, Tehran, Iran; ^2^Division of Gastroenterology and Hepatology, School of Medicine, Johns Hopkins University, Baltimore, MD 21287, USA

## Abstract

The incidence of colorectal cancer (CRC) during pregnancy is so rare. Herein we present a case of colorectal cancer that was missed by pregnancy all over the pregnancy period. The patient was a 37-year-old woman (gravid 4, para 2) referred with the complaints of vaginal discharge and suspicious rupture of membrane (ROM). The patient was pale and the initial physical examination revealed dilation of two fingers, effacement about 30%. She underwent emergent cesarean section which showed adhesions surrounding the uterus, the bladder, and the abdominal wall. Forty days postoperatively, the patient presented with abdominal pain in the left upper quadrant (LUQ). Imaging confirmed a mass in LUQ. Partial colectomy of transverse colon (20 cm) was performed. Postoperative histopathologic study revealed a 7 ∗ 6 ∗ 5 cm mass in transverse colon compatible to stage IIa of the Duck class (T3, N0, Mx). Adjuvant chemotherapy was applied and the patient showed improvements during 7 months followup after surgery. Colorectal cancer in pregnancy is associated with diagnostic and therapeutic challenges which mostly lead to late diagnosis in advanced stages and poor prognosis. A targeted program to improve the general population knowledge and the establishment of a national consultant and screening program particularly for women with a planned pregnancy in the high risk group might be beneficial.

## 1. Introduction

Incidence of colorectal cancer (CRC) during pregnancy is so rare (0.002%) [1], and its common manifestations including abdominal pain, nausea, vomiting, and altered bowel movements are generally found in normal pregnancy [2, 3]. Thus, most of the CRC cases are missed and are diagnosed in advanced stages which are associated with poor prognosis [4]. Here, we present a case of colorectal cancer who was missed by pregnancy all over the pregnancy period and even the labor time.

## 2. Case

The patient was a 37-year-old woman (gravid 4, para 2) who was referred with the complaints of vaginal discharge and suspicious rupture of membrane (ROM). The patient was not aware of her last menstrual period (LMP) and had not any previous sonography result at that time to define gestational age, but she believed that only 5 days remained to the defined date for her elective cesarean section. Her previous pregnancies have led to 2 live boys and one abortion. There was not any remarkable point in his history and the patient did not mention any positive family history of cancer when she was asked. Physical examination revealed neither vaginal bleeding nor decreased fetal movement. The patient was so obese (BMI = 41 Kg/m^2^) and the vaginal examination was not possible due to intolerable pain, thus underwent spinal anesthesia for vaginal examination which revealed a dilation of two fingers, effacement about 30%. Initial laboratory examination showed anemia with hemoglobin (Hb) of 8.9 g/dL and hematocrit of 23.5%. She was advised to perform tubal ligation, but both the patient and her husband did not agree. She underwent emergent cesarean section. During surgery there were lots of adhesions surrounding uterus, bladder, and abdominal wall which were attributed to the previous cesarean sections. 

Postoperatively, the patient was in a good condition without fever and bleeding and was discharged with medical and hygiene instructions. One week after surgery, the patient referred for checkup and removing of the sutures and there was no abnormality except being pallor, thus iron tablet was prescribed.

Forty days after surgery, the patient presented with abdominal pain in the left upper quadrant (LUQ). The patient was severely pale and there was a severe tenderness in LUQ. Physical examination confirmed a mass approximately 4∗5 cm in the LUQ. Abdominal plain X-ray was normal without evidence of remained surgical tools. Abdominopelvic sonography showed a hypoechoic 25∗28 mm mass in the epigastric region anterior to the anteroinferior part of pancreas with fluid concentration and hematoma. Intestinal wall thickness in spleen curvature of colon due to ischemic colitis and limited amount of fluid spreading in intestinal loops were mentioned. The patient was transferred to a general surgery ward. Initial laboratory examination showed severe anemia (Hb: 5.1 g/dL) with raised erythrocyte sedimentation rate (ESR) (73). Other laboratory studies including complete blood count (CBC), coagulation tests, electrolytes, blood urea nitrogen (BUN), creatinine, lactate dehydrogenase (LDH), and indirect coombs were normal or within a normal range. The patient received 6 unite of packed blood cell and Hb levels rose to 11.1 g/dL. Computed tomography (CT) scan confirmed a mass in LUQ, and noted colon changes due to probable surgical complications of recent cesarean section. It was a long time since the surgery and this hypothesis was not acceptable by surgeons. Therefore, patient underwent colonoscopy which showed a mass in a spleen curvature of colon. Patient transferred to the oncology ward and underwent partial colectomy of transverse colon (20 cm). Postoperative histopathologic study revealed a 7∗6∗5 cm mass in transverse colon. The tumor was sharp, extended up to the serosa (T3), without vascular, perineuronal, and lymphoma invasion. None of the 6 removed lymph nodes were involved. Both proximal and distal margins were free of tumors. The tumor should be at least at stage IIa of the Duck class (T3, N0, Mx).

Postoperative reevaluation of the patient defined that the patient had similar transient abdominal pain since the start of her last pregnancy, and was admitted with the impression of threatened miscarriage. She was treated and discharged with decrease in pain intensity. These pains existed all over the pregnancy and were attributed to the pregnancy, previous adhesion, and her obesity. More excitingly, after colonoscopy the patient mentioned that her mother had colon cancer and died due to this reason at age of 60-year-old. At the first interview and all over her previous medical records, this important history was not mentioned. After surgery, the patient underwent adjuvant chemotherapy and she became healthy without any problem 7 months after surgery.

## 3. Discussion

Despite the low incidence rate of 0.07% to 0.1% [5], cancer accounts as a leading cause of death in women in childbearing ages [6]. CRC is among the eight most common malignancies in pregnancy [7]. Although its incidence is rare, it is associated with serious consequences for both the mother and even the fetus [8]. Its clinical manifestation, diagnosis, and treatment options seem a big challenge in front of treating physicians [9–13]. In some cases, it is possible only to save one life between the mother and the fetus which demands a deep ethical consideration and also religious challenges.

Our patient had experienced an intermittent abdominal pain all over her pregnancy with moderate anemia. Common presentations of CRC including abdominal pain, nausea, vomiting, anemia, and rectal bleeding usually masked by pregnancy and it is truly hard to distinguish these findings of what is considered as warning signs of CRC [2, 3, 14]. This delay would lead to late diagnosis of the disease and subsequently poor prognosis. A majority of CRC cases in pregnancy present with Duck class C (44%) in which adjutant therapies are needed to improve the surgical outcome [15]. In our case, appropriate clinical approach to abdominal pain or anemia would help to reach diagnosis. There are limitations to use most of the diagnostic imaging modalities during pregnancy; however, some can be used in specific circumstances. Colonoscopy which is routinely used in nonpregnant women is relatively contraindicated due to the potential mother and fetus risks and also fetal exposure to potential teratogens [16] and only should be preformed with specific cautions. Due to radiation exposure, abdominal CT scan is not recommended specially in the first trimester [17]. Alternatively ultrasonography and magnetic resonance imaging (MRI) could be applied; however, the sensitivity of sonography for detection of micrometastasis is not as high as CT imaging and MRI risks are not fully understood in pregnancy [18, 19].

The main pathogenesis of CRC in pregnancy is still associated with lots of unanswered questions. Some factors including pregnancy hormones, the enzyme cyclocoxygenase-2 (Cox-2), and tumor suppressor protein p53 mentioned to be associated with CRC. A majority of CRC cases have been found to be positive for estrogen (20–54%) [20] and progesterone receptors (10–100%) [21]. Maybe the increased levels of estrogen and progesterone during pregnancy stimulate the growth of tumoral cells with such receptors; however, all reports did not support this hypothesis. Slattery et al. in a study found only one case with positive progesterone receptor among 156 pregnant cases with CRC [20]. The elevated amounts of Cox-2 in CRC patients has raised the hypothesis of its association with colorectal cancer; however, there are little evidences to elucidate its carcinogenic role. These probable genes were not analyzed in our patient. 

Colorectal cancer mostly involves elderly patients and its occurrence in young ages is rare; indeed, there may be some predisposing factors in such patients [22]. Of significance in our patient was the family history of CRC. She was not aware of the importance of this issue in her recent problem. she stated this history after colonoscopy. The lack of general population knowledge in the society and inexistence of an organized screening program even for the most common leading cause of cancer-related deaths is an emergent public health concern for developing countries like Iran [23, 24].

Gestational age and tumor stage are important to select treatment modality. If tumor is resectable, surgical excision is recommended especially in those diagnosed in early pregnancy (before 20 weeks of gestation). In cases of later diagnosis, surgery can be postponed at the earlier possible date at which fetus can be viable (around 32 weeks). In advanced stages, when adjuvant therapy is needed, elective abortion would help to save mother's life, whilst in greater gestational ages it is possible to pursue adjuvant therapy after early delivery. It is important that the mother be fully informed of possible risks of each choice prior to her decision. In religious countries like Iran, there is another extra challenge for parents and clinician, since due to religious beliefs the legal abortion is only permitted up to the week 16 and after this time there would be problems to perform the abortion legally.

CRC is associated with poor prognosis. The median survival in a review of 42 pregnant patients with CRC was less than 5 months and more than half of them (56%) died by the time of the report [25]. Our patient underwent adjuvant chemotherapy and she became healthy without any problem 7 months after surgery.

## 4. Conclusion

Colorectal cancer in pregnancy is associated with diagnostic and therapeutic challenges which mostly lead to late diagnosis in advanced stages and poor prognosis. Targeted program to improve general knowledge and establishing national screening program seem necessary in endemic countries for CRC like Iran. Particularly, women in the high risk group might benefit from the consultant and screening program when they have planned a pregnancy which requires further investigation. 

## References

[1] Girard RM, Lamarche J, Baillot R (1981). Carcinoma of the colon associated with pregnancy: report of a case. *Diseases of the Colon and Rectum*.

[2] Chêne G, Tardieu AS, Favard A, Lebel A, Voitellier M (2006). Colorectal cancer discovered during pregnancy. *Journal de Gynecologie Obstetrique et Biologie de la Reproduction*.

[3] Kömürcü S, Ozet A, Oztürk B, Arpaci F, Altundağ MK, Tezcan Y (2001). Colon cancer during pregnancy. A case report. *The Journal of Reproductive Medicine*.

[4] Nesbitt JC, Moise KJ, Sawyers JL (1985). Colorectal carcinoma in pregnancy. *Archives of Surgery*.

[5] Walsh C, Fazio VW (1998). Cancer of the colon, rectum, and anus during pregnancy. *Gastroenterology Clinics of North America*.

[6] Parker SL, Tong T, Bolden S, Wingo PA (1997). Cancer statistics, 1997. *Ca-A Cancer Journal for Clinicians*.

[7] Doll DC, Ringenberg QS, Yarbro JW (1988). Managment of cancer during pregancy. *Archives of Internal Medicine*.

[8] Mathonnet M, Fermeaux V (2003). Colon cancer in pregnancy. *Journal de Chirurgie*.

[9] Minter A, Malik R, Ledbetter L, Winokur TS, Hawn MT, Saif MW (2005). Colon cancer in pregnancy. *Cancer Control*.

[10] Pigeau H, Dupré PF, Benouna J (2005). Management of rectal cancer in pregnant women. *Bulletin du Cancer*.

[11] Mechery J, Ikhena SE (2007). Cancer of the descending colon during pregnancy. *Journal of Obstetrics and Gynaecology*.

[12] Harma M, Harma M, Uzunkoy A (2005). Colorectal cancer presenting with uncommon soft tissue invasion during pregnancy. *Acta Obstetricia et Gynecologica Scandinavica*.

[13] Chourak M, Tahiri MH, Majbar A (2009). Colorectal cancer discovered during pregnancy. *Gastroenterologie Clinique et Biologique*.

[14] Colecchia G, Nardi M (1999). Colorectal cancer in pregnancy. A case report. *Il Giornale di chirurgia*.

[15] Bernstein MA, Madoff RD, Caushaj PF (1993). Colon and rectal cancer in pregnancy. *Diseases of the Colon and Rectum*.

[16] Cappell MS (1996). Gastrointestinal endoscopy in high-risk patients. *Digestive Diseases*.

[17] Brent RL (1989). The effect of embryonic and fetal exposure to X-ray, microwaves, and ultrasound: counseling the pregnant and nonpregnant patient about these risks. *Seminars in Oncology*.

[18] Nies C, Leppek R, Sitter H (1996). Prospective evaluation of different diagnostic techniques for the detection of liver metastases at time of primary resection of colorectal carcinoma. *European Journal of Surgery*.

[19] Seidman DS, Heyman Z, Ben-Ari GY, Mashiach S, Barkai G (1992). Use of magnetic resonance imaging in pregnancy to diagnosis intussusception induced by colonic cancer. *Obstetrics and Gynecology*.

[20] Slattery ML, Samowitz WS, Holden JA (2000). Estrogen and progesterone receptors in colon tumors. *American Journal of Clinical Pathology*.

[21] Singh S, Sheppard MC, Langman MJS (1993). Sex differences in the incidence of colorectal cancer: an exploration of oestrogen and progesterone receptors. *Gut*.

[22] Isbister WH, Fraser J (1990). Large-bowel cancer in the young: a national survival study. *Diseases of the Colon and Rectum*.

[23] Mousavi SM, Somi MH (2009). Gastric cancer in Iran 1966–2006. *Asian Pacific Journal of Cancer Prevention*.

[24] Malekzadeh R, Derakhshan MH, Malekzadeh Z (2009). Gastric cancer in Iran: epidemiology and risk factors. *Archives of Iranian Medicine*.

[25] Chan YM, Ngai SW, Lao TT (1999). Colon cancer in pregnancy: a case report. *Journal of Reproductive Medicine for the Obstetrician and Gynecologist*.

